# Identification of novel mutations in Mexican patients with Aarskog–Scott syndrome

**DOI:** 10.1002/mgg3.132

**Published:** 2015-02-17

**Authors:** Mariana Pérez-Coria, José J Lugo-Trampe, Michell Zamudio-Osuna, Iram P Rodríguez-Sánchez, Angel Lugo-Trampe, Beatriz de la Fuente-Cortez, Luis D Campos-Acevedo, Laura E Martínez-de-Villarreal

**Affiliations:** 1 Departamento de Genética, Facultad de Medicina y Hospital Universitario “José E. González”, Universidad Autónoma de Nuevo León (UANL)Monterrey, Nuevo Leon, México; 2 Centro Mesoamericano de Estudios en Salud Pública y Desastres, Universidad Autónoma de Chiapas (UNACH)Tapachula, Chis, México

**Keywords:** Aarskog–Scott syndrome, *FGD1* gene, mental retardation, novel mutation, X-linked

## Abstract

Aarskog–Scott syndrome (AAS), also known as faciogenital dysplasia (FGD, OMIM # 305400), is an X-linked disorder of recessive inheritance, characterized by short stature and facial, skeletal, and urogenital abnormalities. AAS is caused by mutations in the *FGD1* gene (Xp11.22), with over 56 different mutations identified to date. We present the clinical and molecular analysis of four unrelated families of Mexican origin with an AAS phenotype, in whom *FGD1* sequencing was performed. This analysis identified two stop mutations not previously reported in the literature: p.Gln664* and p.Glu380*. Phenotypically, every male patient met the clinical criteria of the syndrome, whereas discrepancies were found between phenotypes in female patients. Our results identify two novel mutations in *FGD1*, broadening the spectrum of reported mutations; and provide further delineation of the phenotypic variability previously described in AAS.

## Introduction

Aarskog–Scott syndrome (AAS, OMIM # 305400), also known as faciogenital dysplasia (FGD), is an X-linked syndrome with recessive inheritance, characterized by short stature, hypertelorism, short nose, brachydactyly, and shawl scrotum (Scott 1971; Orrico et al. 2004). Experience in Leuven (Belgium**)** and Manchester (United Kingdom) indicates population prevalence up to 1/25,000 (Orrico et al. 2014). The clinical abnormalities that can be used for diagnosis of AAS are varied; therefore, the criteria described by Teebi et al. (1993) are customarily utilized (Table1). Furthermore, it is difficult to establish carrier status in women, as they may be asymptomatic or show only partial expression of clinical manifestations due X inactivation (Pasteris et al. 1994).

**Table 1:** Clinical description of the Teebi et al. (1993) criteria evaluated in patients and their mothers with AAS.

Features	Patients evaluated								
	Male					Female			
	1	2	3	4	5	6	7	8	9
Primary criteria									
Hypertelorism	+	+	+	+	+	+	+	+	−
Anteverted nostrils	+	−	+	−	+	+	−	+	−
Bottom lip Fold	+	+	+	+	+	+	+	−	+
Brachydactyly/wide fingers	+	−	+	+	+	+	−	+	−
Interdigital tracts	+	−	+	+	+	−	−	+	−
Shawl Scrotum	+	+	−	+	+	NA	NA	NA	NA
Syndactyly	−	−	+	+	−	−	−	−	−
Clinodactyly	+	−	−	−	+	+	−	−	−
Camptodactyly	−	−	−	+	+	+	−	−	−
Short stature	+	+	+	+	+	+	−	+	−
Secondary criteria									
Widow's peak	+	+	+	+	+	+	+	+	+
Ptosis	−	−	−	+	+	−	−	−	−
Downward slant palp	−	−	−	+	+	−	−	−	−
Joint hypermobility	+	+	−	+	+	−	+	+	−
Wide foot	+	−	−	+	+	−	−	−	+
Inguinal/umbilical hernia	−	+	+	−	+	−	−	−	−
Cryptorchidism	+	+	−	+	+	NA	NA	NA	NA
Dysplastic ears	+	+	−	+	+	−	−	−	−
Prominent umbilicus	−	−	+	−	−	−	−	+	−
Additional criteria									
Obesity	+	−	−	+	−	−	−	−	−
Long philtrum	+	+	−	+	+	−	−	+	−
Midface hypoplasia	+	−	+	+	+	+	+	+	−
Dental malocclusion	+	−	+	+	−	−	−	−	−
Transverse palmar crease	+	−	−	−	+	−	−	−	−
Hypospadias	−	+	−	−	−	NA	NA	NA	NA
Frontal bossing	+	+	+	−	−	−	−	+	−
Psychomotor retardation	−	−	−	+	−	−	−	+	−

+, characteristic present; −, characteristic absent, NA, not applicable; ASS, Aarskog–Scott syndrome.

Since the identification in 1994 of mutations in *FGD1* gene in patients with a phenotype consistent with AAS, about 56 different mutations across the 18 exons of the gene have been characterized worldwide (Orrico et al. 2011). The *FGD1* locus is located on the short arm of the X chromosome (Xq11.22) and is essential for normal embryonic development in mammals (Estrada et al. 2001). The *FGD1* protein product is a member of the DBL family, and acts as guanine nucleotide exchange factor of the Rho GTPase Cdc42, inducing a conformational change in the protein that allows interaction with effector proteins that handle a variety of biological processes (Genot et al. 2012). *FGD1* mutations have been identified in approximately 20% of the known cases of AAS (Orrico et al. 2010; Verhoeven et al. 2012). Some studies have found that patients with confirmed molecular evidence have relatively consistent clinical presentations, including hypertelorism, short nose, short and broad hands, shawl scrotum, and a mild to moderate short stature with an acromelic prevalence (Scott 1971; Glover et al. 1993; Orrico et al. 2010).

Previous reports of molecularly proven cases have all corresponded to Caucasian patients, and the majority of mutations identified to date are unique within each family. Furthermore, no hotspot or common mutations exist that have a demonstrated genotype–phenotype correlation. Therefore, the purpose of this study was to clinically and genetically characterize four nonconsanguineous Mexican (non-Caucasian) families with an AAS syndrome phenotype. This led to the identification of two novel mutations in *FGD1*, representing 60% of the patients in these families (Table1).

## Materials and Methods

### Patients and sample collection

A clinical and dysmorphologic evaluation was performed on patients by applying the Teebi criteria (Teebi et al. 1993) to duos (mother/son) in four nonconsanguineous families with an AAS syndrome phenotype who received care at the Department of Genetics, “Dr. José Eleuterio González” University Hospital, the Autonomous University of Nuevo León. After written informed consent was obtained from patients, peripheral blood samples were collected, and total genomic DNA was extracted using the QIAamp® DNA Mini and Blood Mini extraction kits (Qiagen, Valencia, CA) according to the manufacturer's instructions. The study was approved by the ethics committee of the same institution.

### Sequencing and mutation detection

PCR amplification for all *FGD1* exons (NM_004463.2) was performed using the primers previously described by Orrico et al. (2010). The PCR products were sequenced bidirectionally using the same primers and BigDye version 3.1 (Applied Biosystems, Foster City, CA) according to the manufacturer's instructions. After purification, products were run on an ABI Prism 3130 Genetic Analyzer (Applied Biosystems). The results were analyzed by visual inspection using Geneious 4.7.6 software (Biomatters, Auckland, NZ).

## Results

**Figure 1:**
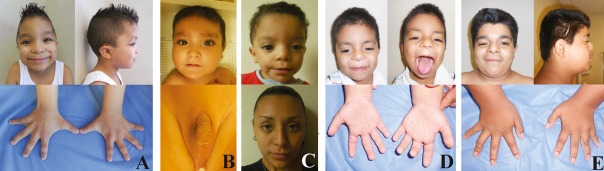
Patients with clinical features of Aarskog syndrome. (A) Patient 1; note distinctive facial characteristics and interdigital tracts in both hands. (B) Patient 2; discrete facial features and the shawl scrotum can be appreciated. (C) Patient 3 and his mother (patient 8); note prominent forehead, widow's peak, hypertelorism, and fold under the lower lip. (D) Patient 4 with widow's peak, midface hypoplasia, ptosis, clinodactyly, and brachydactyly. (E) Patient 5, brother of patient 4, with distinctive facial features, clinodactyly, and brachydactyly.

Four families were recruited with an AAS phenotype, from which nine samples were obtained from affected individuals: five males and four females, with mean age of 6 and 31 years, respectively. The clinical criteria utilized for diagnosing patients with AAS Syndrome are shown in Table1. During clinical evaluation, the following features were more prevalent in men: short stature, hypertelorism, and a fold of the lower lip were observed in 100% of patients; brachydactyly, interdigital tracts, and shawl scrotum were also seen in 80% of patients. Secondary criteria included a widow's peak, present in 100% of patients, followed by cryptorchidism, dysplastic ears, and joint hypermobility in 80% of patients. Finally, among the additional features described, long philtrum and mid-facial hypoplasia were present in 80% of patients, followed by poor dental occlusion and frontal bossing, present in 60% of patients. Photographs of some patients with the observed clinical characteristics are shown in Figure1.

We showed two nonsense mutations (p.Gln664* and p.Glu380*) both inherited by each mother. The results of molecular testing are presented in Table2. In summary, a recognizable *FGD1* mutation was identified in only 60% of individual who were analyzed.

**Table 2:** Changes found in cDNA and its effect on the protein.

Case	Gender	Clinical criteria	gDNA	cDNA	Protein	Type of mutation
1	M	19	g.54495273C>A	c.1138G>T	p.Glu380^*^	Novel
2	M	12	–	–	NC	–
3	M	13	–	–	NC	–
4	M	20	g.54481906G>A	c.1990C>T	p.Gln664^*^	Novel
5	M	20	g.54481906G>A	c.1990C>T	p.Gln664^*^	Novel
6	F	9	g.54495273C>A	c.1138G>T	p.Glu380^*^	Novel
7	F	7	–	–	NC	–
8	F	11	–	–	NC	–
9	F	3	g.54481906G>A	c.1990C>T	p.Gln664^*^	Novel

gDNA, genomic DNA; cDNA, complementary DNA; M, male; F, female; NC, no change.

## Discussion

Clinically, patients with AAS present with various abnormalities (Teebi et al. 1993). In this study, several primary characteristics were observed, namely, short stature, brachydactyly, shawl scrotum, hypertelorism, and a fold below the lower lip (Scott 1971; Orrico et al. 2004). Mutations of *FGD1* gene represent the etiology of AAS (Orrico et al. 2010; Verhoeven et al. 2012). Various types of *FGD1* mutations have been identified, ranging from point mutations to large deletions distributed throughout the gene; most result in a truncated protein (Orrico et al. 2000; Hou et al. 2003; Orrico et al. 2011; Volter et al. 2014). In this study, *FGD1* mutation detection was conducted in four Mexican families; two novel mutations were identified in two no Caucasian families with AAS (c.1138G>T and c.1990C>T); in each case, these produce a stop codon leading to a truncated protein. These two mutations arose in different domain types of the *FGD1* protein: Dbl homology/RhoGEF activity (DH); and pleckstrin homology (PH).

**Figure 2:**
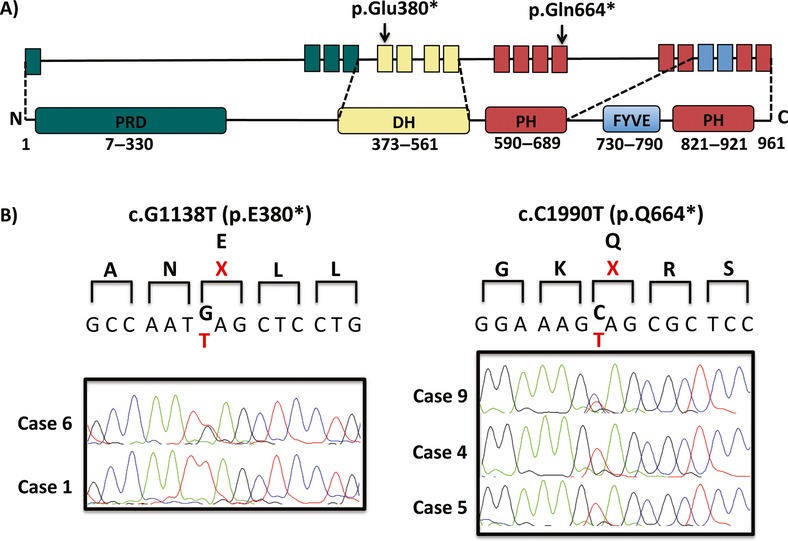
Detection of *FGD1* mutations. (A) Schematic representation of the domains of the *FGD1* protein showing mutations (p.Glu380* and p.Gln664*) identified in patients with AAS. Arrows indicate the positions of the mutated nucleotides in *FGD1*. (B) sequencing results (p.Glu380* and p.Gln664*) detected in exon 5 and 12, respectively. The altered amino acids are shown in red.

The c.1138G>T (p.Glu380*) variant, a previously unreported mutation identified in exon 5 that produces a stop codon, is located within the *FGD1* DH domain (Fig.2), which has been shown to play an important role in regulating cell growth and differentiation (Pasteris et al. 1994). Interestingly, the residue 380 was already detected as involved in mutational events, although the type of mutation is missense (p.E380A) (Orrico et al. 2004). This mutation was identified in the mother and child (patients 1 and 6). Patient 1, a 6-year-old boy evaluated due to short stature at 2 years of age, had eight primary, clinical criteria identified (Fig.1A), while his mother, patient 6, had attenuated features of this syndrome.

The variant c.1990C>T (p.Gln664*), identified in exon 12 of *FGD1*, is also a novel mutation that produces a stop codon. This mutation disrupts the coding sequence of the PH domain (amino acids, aa, 590–689; Fig.2). It is widely accepted that the majority of proteins with domains in the PH family are involved in the phosphorylation of inositol phospholipids. They play a central role in a number of cell processes, ranging from intracellular signal transduction in the plasma membrane, actin cytoskeleton organization, and prevention of apoptosis, to regulation of vesicular endocytosis (Orrico et al. 2000). The mutation was identified in two siblings (patients 4 and 5) and their mother (patient 9). Patient 4, at 12 years of age had the most phenotypic characteristics of AAS of all the patients reported in this study, as defined by Teebi et al. (1993), and shared the vast majority of clinical features with his younger brother of 4 years of age (patient 5). Interestingly, patient 4 also displayed delayed learning, a finding that may not yet have been evident in his brother. Delayed learning has been observed in other cases (Lebel et al. 2002; Orrico et al. 2004), although apparently has not reported genotype–phenotype correlation that can predict whether a patient will have delayed learning, even this feature is considered rare in AAS. The boys’ mother, who was heterozygous for the mutation, phenotypically only, displayed a “widow's peak” and wide feet as unique features compatible with AAS.

The failure to identify pathogenic mutations in *FGD1* in the other two families (patient 2 and mother, patient 7; patient 3 and mother, patient 8) can be attributed largely to the clinical heterogeneity of AAS being as a clinical and not a molecular diagnosis, meaning that clinical heterogeneity and the lack of identifiable mutation do not exclude the diagnosis. Both patient 3 and his mother had sufficient clinical findings to support the diagnosis; however, a molecular basis for the disorder in this family cannot be ruled out. A recent publication documents, for example, the occurrence of a mutation in the branch point of exon 13 by exome sequencing, that conditions premature termination of translation due to a skipped exon (Aten et al. 2013); making it important to consider additional molecular diagnostic technologies. However, the differential diagnosis must also consider other disease such as Noonan syndrome, SHORT syndrome, and Robinow syndrome, as their pathologies overlap with many clinical features of AAS as short stature, long philtrum, micrognathia, hypertelorism, broad nasal bridge, anteverted nostrils, cryptorchidism, brachydactyly, clinodactyly, small hands, ptosis, skeletal and genitourinary abnormalities (Orrico et al. 2011; Tartaglia et al. 2011).

Whereas the best characterized form of AAS is associated with mutations in *FGD1*, only about 20% of disease prevalence can be explained by this mechanism, and the genetic cause has not yet been identified in most families. Therefore, it is believed that other genes may be involved in the etiology of this disorder. GEFs comprise a large family of regulatory proteins with over 21 having been identified across the human genome to date. GEFs control various intracellular processes such as gene expression, rearrangements of the cytoskeleton, intracellular trafficking, and metabolism (Hall 2005; Jaffe and Hall 2005; Gupta et al. 2013). For example, *in vitro* studies have shown that *FGD4* and Vav1 activate signaling downstream from Cdc42 as does *FGD1*; and that these proteins play critical roles in the formation of the actin cytoskeleton and embryonic development (Olson et al. 1996). Therefore, a form of autosomal recessive inheritance, with similar underlying mechanism to the X-linked variety, cannot be ruled out for this disorder.

Clinical examination of the mothers of the probands showed that four women between 28 and 37 years old found phenotypic heterogeneity in most cases. Dramatic similarity was observed between patient 6 and her son, patient 1. Patient 6 was the mother with the most primary clinical features, sharing the same, albeit attenuated, phenotypic traits with her child, with the exception of interdigital tracts; but not sharing the secondary and additional characteristics, of which she shared only the “widow's peak” and midface hypoplasia. Molecularly, the same mutation was found in both patients. As has been described elsewhere, the carriers of a mutation may exhibit an attenuated phenotype AAS (Mikelsaar and Lurie 1992; Pasteris et al. 1994); therefore, behaves as an X-linked recessive disorder, with phenotypic attenuation in females likely due to wild-type *FGD1* expression from the second X chromosome (Park et al. 2010). It is further possible that other genes or regulatory factors may modulate *FGD1* expression in females, moderating the phenotype (Gao et al. 2011; Zou et al. 2011).

The finding in this study of two separate mutations within *FGD1* is consistent with previous studies, wherein each identified mutation was unique to each family (Orrico et al. 2010), with the exception of mutations c.528insC, p.R656X, and p.R443, which were reported in several unrelated families (Al-Semari et al. 2013). However, these families do not share general clinical characteristics, nor is there any evidence suggesting that the mutation influences the clinical phenotype of the disease.

In conclusion, this study represents the first molecular analysis of the *FGD1* gene in Mexican patients with characteristic AAS phenotypes, and presents the discovery of two novel mutations not previously reported in the literature. The main features observed in our probands were short stature, hypertelorism, a lower lip fold, and a widow's peak, confirming the variability of phenotypic expression described in previous publications. Our data demonstrate that even when the clinical criteria dictated by Teebi et al. (1993) are met, conventional sequencing techniques are only able to identify recognizable mutations in *FGD1* in a few of families. Numerous other possible causes for AAS, such as deletions or intronic mutations of *FGD1*, are not detectable by this technique. Finally, the results obtained in this study allowed us to provide more accurate genetic counseling for the women identified as mutation carriers, as opposed to relying upon the general familial recurrence risk.
